# Community perceptions of health insurance and their preferred design features: implications for the design of universal health coverage reforms in Kenya

**DOI:** 10.1186/1472-6963-13-474

**Published:** 2013-11-12

**Authors:** Stephen Mulupi, Doris Kirigia, Jane Chuma

**Affiliations:** 1 KEMRI-Wellcome Trust Research Programme, P.O. Box 230, Kilifi, Kenya; 2 Centre for Tropical Medicine, Nuffield Department of Clinical Medicine, University of Oxford, Oxford, UK; 3 School of Economics, University of Nairobi, Nairobi, Kenya

## Abstract

**Background:**

Health insurance is currently being considered as a mechanism for promoting progress to universal health coverage (UHC) in many African countries. The concept of health insurance is relatively new in Africa, it is hardly well understood and remains unclear how it will function in countries where the majority of the population work outside the formal sector. Kenya has been considering introducing a national health insurance scheme (NHIS) since 2004. Progress has been slow, but commitment to achieve UHC through a NHIS remains. This study contributes to this process by exploring communities’ understanding and perceptions of health insurance and their preferred designs features. Communities are the major beneficiaries of UHC reforms. Kenyans should understand the implications of health financing reforms and their preferred design features considered to ensure acceptability and sustainability.

**Methods:**

Data presented in this paper are part of a study that explored feasibility of health insurance in Kenya. Data collection methods included a cross-sectional household survey (n = 594 households) and focus group discussions (n = 16).

**Results:**

About half of the household survey respondents had at least one member in a health insurance scheme. There was high awareness of health insurance schemes but limited knowledge of how health insurance functions as well as understanding of key concepts related to income and risk cross-subsidization. Wide dissatisfaction with the public health system was reported. However, the government was the most preferred and trusted agency for collecting revenue as part of a NHIS. People preferred a comprehensive benefit package that included inpatient and outpatient care with no co-payments. Affordability of premiums, timing of contributions and the extent to which population needs would be met under a contributory scheme were major issues of concern for a NHIS design. Possibilities of funding health care through tax instead of NHIS were raised and preferred by the majority.

**Conclusion:**

This study provides important information on community understanding and perceptions of health insurance. As Kenya continues to prepare for UHC, it is important that communities are educated and engaged to ensure that the NHIS is acceptable to the population it serves.

## Background

Many low and middle income countries (LMICs) are currently considering how to reform their health care systems to provide effective financial risk protection for all, as part of universal health coverage (UHC). Universal health coverage is defined as a situation where the whole population has access to appropriate promotive, preventive, curative and rehabilitative health care when they need it and at an affordable cost. It has two main goals: financial risk protection and access to needed care [1]. Universal health coverage also includes objectives related to equity in access, quality services and broader social protection. The 58^th^ World Health Assembly urged member states to “ensure that health-financing systems introduce or develop prepayment of financial contributions for the health sector, with a view to sharing risk among the population and avoiding catastrophic health-care expenditure and impoverishment of individuals as a result of seeking care” [2]. Following on this call, the World Health Report for 2010 focused on UHC and identified the important role played by health systems financing in making progress towards this goal [1], and in 2011, the 64^th^ World Health Assembly re-emphasized the urgency of implementing sustainable health financing structures and the need to monitor progress towards achieving UHC. Several other initiatives have been put forward to support progress towards UHC.

Health systems in many low and middle-income countries have been funded predominantly through out-of-pocket (OOP) payments. The negative implications of OOP payments are well documented: they impact negatively on the demand for health care, contribute towards household poverty, promote inequities and generate little revenue [3-10]. This evidence has contributed to a recent shift in health financing debates worldwide; away from OOP payments towards mechanisms that protect the population from catastrophic costs and impoverishment. Such mechanisms include health systems that are funded through general taxation revenues and/or health insurance contributions that pool risks and promote access to care for all irrespective of their socio-economic status [2,11]. In many African countries, including Kenya, health insurance schemes (either on a voluntary or mandatory basis) are being promoted as the main sources of health funds to support rapid progress towards UHC. However, the concept of health insurance is relatively new in many African countries, is hardly well understood, and it remains unclear how this type of health care funding will function in countries where the majority of the population work outside the formal sector.

Kenya is one of the countries where advanced plans for UHC have been made. The Kenya health financing strategy, the constitution and the country’s vision 2030 highlight UHC as central to the country’s development and commit to its achievement by 2030 [12-14]. The health financing strategy is built around the principles of solidarity, where income and risk cross-subsidisation will play a major role; responsibility, ensuring that the health system puts people first and that health care providers offer quality services and promote efficiency; equity, where all Kenyans will have access to a basic package of health services according to their need and; transparency, which involves ensuring that purchasers, providers and users have access to information regarding the operations of the system [13]. Central in the health financing strategy and policy debates in Kenya is the role of different health insurance mechanisms in generating additional revenue for the health sector and offering financial risk protection. Initial attempts to introduce a mandatory health insurance in 2004 were met with resistance from different stakeholders. The social health insurance bill was passed in parliament but the president declined to sign it due to a mix of both technical and political reasons. The process of searching for the best approaches to UHC was again initiated in 2007 and continues to date. Central to ongoing discussions is the role of health insurance in UHC and how a national health insurance scheme (NHIS) can be designed to ensure that UHC goals are achieved and sustained. The proposed study aimed to contribute to this process by exploring communities’ understanding and perceptions of health insurance and their preferred designs for a NHIS. The study explores issues related to premium payment levels and other critical design features of a NHIS, but it does not attempt to elicit willingness-to-pay (WTP) or ability-to-pay explicitly. Communities are the major beneficiaries of any UHC reform. It is important that Kenyans understand the implications of new financing mechanisms and that their preferred design features are put into consideration to ensure acceptability and sustainability.

### Overview of health financing in Kenya

The Kenyan health system relies heavily on OOP payments charged in both public and private health facilities. Out-of-pocket payments accounted for 51.1%, 35.9% and 24.5% of the total health expenditure in 2001, 2005 and 2009 respectively [11,15,16]. The majority of the population cannot afford to pay for health care, the poor are less likely to utilize health services when they are ill, and wide disparities in utilization exist between geographical regions and urban and rural areas [17,18]. Health insurance coverage is relatively low in Kenya. Only 10% of the population have some form of health insurance cover. The National Hospital Insurance Fund (NHIF), a mandatory health insurance fund covering public and private formal sector workers and their dependents is the main health insurer. Those working outside the formal sector can join the NHIF on a voluntary basis. Coverage among formal sector workers is about 99%, but efforts to reach the informal sector workers have not been successful. The NHIF covers about 2.7 million people; of these only 600,000 work outside the formal sector. The NHIF provides comprehensive cover for inpatient services in public and faith-based facilities. Private facilities accredited by NHIF are paid through a flat daily rate, based on the range of facilities available including X-rays, intensive care unit, number of health personnel, laboratories, operating theatres, overall area occupied, number of wards and ambulances; facilities are therefore paid different levels of rebate, depending on their accreditation score [19]. The NHIF is being considered as a potential institution to be transformed to a NHIS, as part of UHC.

Community-based Health Insurance Schemes (CBHIs) are not widespread in Kenya. According to the Kenya Community Based Health Financing Association (KCBHFA), there are about 38 registered CBHIs in Kenya, although it is not clear how many are still active. CBHIs cover about 470,550 beneficiaries, approximately 1.2% of the Kenyan population. The benefits packages provided by CBHIs differ, but mainly involve inpatient care in selected public and faith-based health facilities. The CBHIs identify specific health care facilities in close proximity to their geographical locations to provide health care services to their members. A few CBHIs are linked to the NHIF, where community members are covered by the NHIF and a CBHI. Under such an arrangement, the CBHI cover is used to meet the costs of services that are not covered through the NHIF, for example surgery in faith based facilities and the costs of buying prescribed medicines and other essential supplies when these are not available in public health facilities.

## Methods

### Study setting

Data were collected in Nyeri and Kirinyaga districts located in the Central part of Kenya. The two districts were chosen because they have a long history of CBHIs, covering around 200,000 people and their dependants. Coffee and tea are the main cash crops and sources of income in both districts. Data were collected from 6 villages (3 from each district); selected randomly from a list of villages where CBHIs were in existence. Village leaders’ views on the selected sites were also sought to ensure that there was buy-in for the study, to maximise participation and ensure safety of research staff, since some of the regions were prone to violence at the time of the study.

### Data collection methods

Data presented in this paper are part of a wider study that explored feasibility of health insurance as a mechanism to address health system inequities in Kenya. Quantitative and qualitative data collection methods were used to explore the variables of interest. Data were collected through a cross-sectional household survey (n = 594 households) and focus group discussions (n = 16). Survey households were selected through two stages. First two districts were selected from a list of districts with high CBHI coverage, following discussions with the KCBHFA. A list of villages where CBHIs operate was made, and 3 villages (clusters) were selected per district. All households in the selected villages were mapped and given a unique identification number. A total of 100 households per village were then randomly selected from a complete list of households. All selected households participated in the survey regardless of whether they belonged to a health insurance scheme or not. Questionnaires were administered to household heads or their spouses, and in their absence, another senior household member. Call-back visits were made to 20 randomly selected households in each district to verify data quality. Data were collected on self-reported illness, health care utilisation patterns, health care payments, knowledge of health insurance in general, the NHIF and preferred designs for a future NHIS.

The 16 focus group discussions (FGDs) were conducted in 4 of the 6 villages in which the household survey had previously been conducted. Two villages were dropped due to security concerns. The FGDs were equally distributed across the villages and were conducted separately with insured (n = 8) and uninsured population (n = 8). Conducting FGDs separately for the insured and uninsured population was important to ensure that those who were already members of health insurance scheme did not dominate the discussions and bias the findings as they would be expected to have better understanding of health insurance compared to their uninsured counterparts. Separate FGDS were conducted with men and women to ensure that participants were comfortable expressing their views. Efforts were also made to ensure that group participants belonged to a similar age-group. FGDs discussions were audio-recorded and detailed field notes taken.

Key topics explored in the FGDs include: knowledge and understanding of health insurance, membership of schemes, reasons for joining schemes, and preferred designs of a national health insurance scheme including revenue collection and pooling agencies, benefit packages and purchasing arrangements.

### Data analysis

Cross-sectional household survey data were double-entered into Visual FoxPro version 9.0, and analysed using STATA version 11 to generate descriptive statistics. Qualitative data analysis was done using the thematic framework approach. FGD interviews were transcribed verbatim and typed into Microsoft Word. The researchers read through the transcripts several times to familiarize themselves with the data, to code and identify sub-themes and themes. The first step involved developing a thematic framework based on the information on the interview guides. The thematic framework was then revised to include new themes that emerged from the data, and which were not easily identified from the topic guide. After constructing the framework, themes were indexed and the various segments of the transcripts sorted into the relevant categories using NVivo 8 software.

### Ethical approval

Ethical approval was obtained from the Kenya Medical Research Institute (protocol no. 1609).

## Results

### Socio-demographic characteristics

Table 1 presents an overview of the socio-demographic and economic characteristics of the population. A total of 594 households participated in the cross-section survey, which yielded 2419 individuals. Mean household size was four and ranged from 2 to 14. Most people were aged below 35 years (60.1%). About 41% of adults had secondary level education; 46.1% had at least some level of primary school education; and 8% had been educated to tertiary level. Only 5% of adults had never attended school. The majority of the residents were subsistence farmers (69.4%). Other income generating activities included formal employment (6.6%) and small scale businesses (6%).

**Table 1:** Socio-demographic and economic characteristics of survey households

**Variable**	**Number (%)**
Households interviewed	
• Nyeri	289 (48.7)
• Kirinyaga	305 (51.3)
Total	594 (100)
Number of individuals	
• Male	1190 (49.2)
• Female	1229 (50.8)
Total	2419 (100)
Highest education level among adults	
• Primary school	718 (46.1)
• Secondary school	639 (41.0)
• Tertiary	123 (7.8)
• None	79 (5.1)
Total	1559 (100)
Main occupation for adults	
• Subsistence farming	1080 (69.4)
• Formal employment	103 (6.6)
• Businesses	94 (6.0)
• Other	280 (18.0)
Total	1557 (100)

### Membership of health insurance schemes

About half of the households (52.9%) had at least one member in a health insurance scheme (Table 2). This amounted to 41% of all individuals enumerated in the household survey. The NHIF was the main insurer (41.1%), followed by community based health insurance schemes operating in the study setting (36.2%). About 14% of households had membership of both a CBHI and NHIF. Most households had been members of a health insurance for more than two years (59.5%); 17.6% had been members for a period greater than one year, but less than two years, while 22.9% had membership for less than one year. The main motivation for enrolling into a scheme for those households that had health insurance coverage was to ease access to health care by reducing costs (77.9%). Contributions were mainly paid annually (65.9%) or monthly (34.1%). Mean monthly contribution rate was Kenya Shillings (KES) 193 (US$ 2.4) (median = KES 160(US$ 2). Most CBHIs packages allowed members to receive services from both public and private facilities (66.3%), 23.8% covered treatment in public facilities only, while 5.5% only purchased services from faith-based organisations.

**Table 2:** Membership of health insurance schemes

**Variable**	**Number (%)**
Household with at least one member in health insurance	314 (52.9)
Number of individuals with insurance cover	991 (41.0)
Type of insurance coverage	
• NHIF	407 (41.1)
• CBHI	359 (36.2)
• Both CBHI and NHIF	139 (14.0)
• Other	86 (8.7)
Total	991 (100)
Period of membership	
• < 6 months	32 (8.9)
• 6–12 months	50 (14.0)
• 13–24 months	63 (17.6)
• >24 months	213 (59.5)
Frequency of contribution	
• Monthly	122 (34.1)
• Annually	236 (65.9)
Monthly contribution rates in Kenya Shillings (US$)	
• Mean contribution	193 ($ 2.42)
• Median contribution	160 ($ 2.00)
Reasons for not belonging to health insurance	
• Cannot afford	135 ( 43.5)
• Unawareness of schemes existence	67 (21.6)
• Not compulsory	26 (8.4)
• Do not trust the schemes	20 (6.5)
• Other	62 (20.0)
Total	310 (100)

Key reasons for not enrolling in the schemes were high contribution rates, which were deemed unaffordable to many (43.5%) and unawareness about schemes existence and the services they offered (21.6%). About 14.6% of people who did not belong to any health insurance scheme at the time of the survey had previously been members of an insurance scheme. Of these, 72.5% had been members of the schemes for more than two years. Dropout rates were mainly attributed to lack of funds to renew annual membership (45.5%), loss of job or retirement, in cases where membership was linked to employment (36.4%) and inadequate benefits package (11.4%).

### Knowledge, awareness and understanding of health insurance

The majority of individuals (77%) who did not belong to any form of health insurance reported that they were aware of at least one CBHI operating within the community. Of these, 55.7% reported that they were aware of the procedures required to join a CBHI. Results from FGDs showed mixed patterns regarding awareness and understanding of health insurance. In most FGDs, participants expressed lack of awareness of health insurance and attributed this to limited efforts to promote CBHIs. Others felt that being a rural setting, people hardly travelled to urban areas, where such information was readily available, and therefore knew very little about health insurance in general and its role in health care payments. It was reported that those who were likely to have a good understanding of health insurance were people in formal employment because it was mandatory for them to be members of the NHIF and/or CBHIs.

“There are people who know about health insurance, but it is a very small percentage. Those who do not know are not ignorant…, but there has been no attempt to have a comprehensive campaign to create awareness. The few who know about health insurance are those who have travelled and become exposed to this information. Most of the people here are farmers and are not very exposed. The ones who are aware are mostly those who are formally employed. Due to being at work, they do not have the time to share [information about health insurance] with the other people.” (FGD 14–215,27; old male non-members).

Others felt that officials of the CBHIs were trying to educate people about the schemes through various channels, including churches, funerals and other social gatherings, but this information did not always reach everyone.

“I have heard most of this information in the Catholic Church. I think the group [CBHI] must have originated from there and promoters are there almost every other Sunday. That is all I know since I am not a CBHI member.” (FGD 14–217, 4; old male non-members).

“I can say that I came to learn about the CBHI about a week ago when I was invited to be in the funeral organising committee of a boy who had died. As we were worrying about where to get money for the hospital bill since the boy had been ailing for some time, […] CBHI official told us not to worry about the hospital bill as the boy was insured by his grandmother who is a member of Ugima wa Mwiri [a CBHI].” (FGD 6–99, 29; young male members).

There was a good understanding of the role of health insurance, but it was not always clear how health insurance schemes function, with many equating them to merry-go-rounds (rotating savings groups, that are very common among women). Most people reported that the main aim of health insurance is to help members to meet costs of treatment when they fell ill, and that this was important because of the uncertainty associated with illnesses and the high costs of treatment. The role of health insurance was described in various ways: being covered for healthcare when ill; saving money for illness; and protecting oneself from unexpected events. In most cases, respondents linked their understanding of the role of health insurance to their past experiences, or those of their friends and relatives:

“I had my brother who was admitted in hospital and I was told if he had been a CBHI member, they would have cleared his bill.” (FGD 4–63, 30; old male non-members).

### Factors influencing membership of health insurance schemes

The majority of FGD respondents who were already enrolled in health insurance schemes reported that scheme membership was open to all regardless of socioeconomic status. However, joining the scheme depended on an individual’s willingness, preferences and ability to pay. People of high socioeconomic status and those who are in formal employment were more likely to join health insurance schemes compared to the rest of the population.

“I think some households do find it easier to join CBHIs than others. For example when my wife was employed she joined an insurance scheme and she told me that I was also a beneficiary. I do not think I would ever have joined an insurance scheme and did not even ask for details. So an employed person may find it easier to join. At the same time those who produce more tea are more likely to instruct that money for health insurance be deducted from their income. So in my view those without good income are unlikely to register in such groups.” (FGD 10–165, 17; young male non-members).

CBHI members emphasised that while the schemes were open to all, they were specifically targeting the poorest populations. However, none of the schemes had any arrangements to waive or subsidise contributions for the poorest groups, although it was reported, that CBHIs had different packages to care for people of different socio-economic status: a package costing KES 2,300 per year (approximately US$ 28.8) and another costing KES 500 (US$ 6.3). Access to benefits largely depended on the package, with those contributing more, enjoying more benefits.

“You see that is why they came up with different packages so that all socio-economic groups are catered for. For example, some time back I could hear my brother who was employed discussing about amenity wards [private wards in government hospitals] where a patient can have their own room. I can compare those who pay KES 2,300 (US$ 28.8) to those who could use amenity wards while those paying KES 500 (US$ 6.3) […] are the ordinary people.” (FGD 13–206,24; old female members).

“As someone else said it goes with the package one is able to pay which in turn depends on financial status. Like me I pay KES 500 (US$ 6.3) according to my ability. Another person is better off and is able to pay the package that allows him or her go to more expensive hospitals. If my financial ability allows me to pay for the Karatina District Hospital package [public hospital], that is where I will go.” (FGD 7–115, 25; young female members).

Other factors that made it easy for people to belong to health insurance schemes were: affordable contribution rates and favourable contribution mechanisms, where members were allowed to make their contributions in instalments or having them linked to agricultural produce like tea and coffee and their contribution deducted directly from their earnings during the peak payment period (usually once or twice a year). Many also expressed confidence in leaders of the schemes for proper management of finances and monitoring of healthcare services rendered to the members by the accredited providers to ensure that the members are offered quality services. For some, ill health was a motivation for belonging to a scheme, while others did not see the reason of making contributions towards health insurance when they were in good health.

“The reason why some people joined the schemes is because they or their family members were ailing. Those who are healthy do not join health insurance. Now you are telling us to contribute this money and we do not see who is receiving treatment but it is for keeping aside for the future… that cannot work.” (FGD 4–73, 18; old male non-members).

“There is also something else I see the people consider. Some people may also for instance see that a member of the family has an illness that is deteriorating with time. You find such a person seeking to join the CBHI. I do not know how they manage to pay but I have observed that such people strive to join probably because they know the burden may become overwhelming.” (FGD 6–4; 129 young male non-members).

Non-members and drop-outs had different opinions about the schemes and reasons for not belonging. While the members reported that contribution rates were well linked to peak agricultural seasons, allowing many people to meet the deadlines, non-members reported that this was not the case for most CBHIs. For example, the month of July was reported to be financially difficult for those relying on tea produce as their main source of income, yet this was the month when some schemes expected them to make payments. Not being able to pay in instalments was another reason given that made it difficult for people to join CBHIs. Limited understanding of health insurance prevented people from becoming members. Some expected their contributions to be refunded by end of the year in case they did not get sick or have the same forwarded to the next financial year. Yet, others viewed them as savings that should attract interest and considered it a waste of opportunity contributing money that ‘just lay idle’ for the duration that they were in good health and therefore could not benefit from the scheme.

“…if they make contributions for a year and they do not get ill, they start thinking that they are just helping other people and therefore stop paying.” (FGD 2–33,7; young male members).

Perceived poor quality of services at accredited facilities, inadequate benefit packages and high co-payments also hindered people from joining health insurance schemes and/or contributed to drop out rates. Poor service provision was exemplified by lack of laboratory equipment and x-ray machines, long waiting times, corruption (and conflict of interest), and discrimination of patients according to scheme membership or perceived socioeconomic status. Other complaints included poor hospitality, including rude hospital staff and inadequate ward facilities (overcrowded wards, inadequate bedding, worn-out patient uniforms) and poor diet.

“…The hospitals that CBHIs work with [meaning those accredited to provide services to CBHI members]. As a member you find you are not being given good service as compared to someone who is paying cash. This may make it difficult to join the CBHI. The doctors there just ignore card holders [CBHI members]. It is important for officials to monitor quality of services so that people can be encouraged to join the schemes”. (FGD 11–181, 20; old female non-members).

“They do not provide good care to patients especially when they know you are a CBHI member. You are expected to buy the drugs you will use or they can also tell you they will order for you some drugs…but the drugs belong to the hospital…they tell you they are giving you drugs that belong to another patient and you are expected to pay later on.” (FGD 2–25, 26; young male members).

“Yes, the CBHI helps people when they are admitted in the hospital but at times one may become ill and is prescribed for some drugs, needs to have an X-ray taken and other tests….and all that is not covered by the health insurance schemes. We find this to be burdensome and we would like the CBHIs to cover all this…., it would be very helpful.” (FGD 1–3, 20; old male members).

Health insurance schemes were perceived to have many advantages by both members and non-members, including offering financial protection to members, making members feel at ease when their relatives were in hospitals and building on solidarity to help other community members. It was reported that in the future, community members would be reluctant to contribute towards helping families to clear hospital bills due to the harsh economic conditions, and health insurance will be the only way of ensuring that such people can pay for health care.

“Yes, it is good because if you do not have money and a family member is admitted, the CBHI will pay the bill and hence the patient will not be detained in hospital. It is good.” (FGD 11–180, 24; old female non-members).

“For instance, in Tumutumu hospital [faith-based facility], the hospital bill may run into thousands of shillings forcing such people to sell their cattle and other assets. That is why people join health insurance schemes because even when an illness strikes, a member will not feel anxious about the hospital bill.” (FGD 2–18, 6; Young male members).

“I too have not been a beneficiary but what makes me happy is the knowledge that I am fulfilling God’s wish to help a person who is ill….we are fulfilling some of God’s wishes including helping one another, acts of mercy and just giving someone hope.” (FGD 12–192, 11; young male members).

### Communities’ perceptions and understanding of the National Hospital Insurance Fund

The majority of household survey respondents knew that the NHIF existed in the country (91.2%). However, only 66.7% were aware that membership of the NHIF is open to those working outside the formal sector. Information about NHIF was mainly passed through relatives who belonged to CBHIs (44.8%) and the media (21.1%). Only 51.1% of respondents reported knowing the procedures for enrolling in the NHIF but 57.7% did not know what the contributions were. A large proportion of non-NHIF members expressed their willingness to join the scheme (64.2%), but people were concerned that the current contribution of KES 160 (US$ 2.0) per month for people working in the informal sector was unaffordable to many.

Results from FGDs revealed similar levels of NHIF awareness and concerns about affordability of premiums and benefit packages. Most people were of the opinion that only those working in the formal sector could belong to the NHIF due to the expensive nature of the premiums, although it was reported that those working outside the formal sector are increasingly getting to know more about the NHIF and choosing to be members. Concerns were also expressed regarding the NHIF, with people reporting that it only caters for the wealthy population, who are the minority.

“Let us say that it is now that the people are realising that they can join the NHIF. Before most people used to think that only those who are employed can join.” (FGD 13–212, 2; old female members).

“We have heard about it but it is only the rich who join. You are asked for money when you cannot even make ends meet, then where you will get the money. If someone asks you for two thousand shillings (US$ 25.0) when the last time you had a thousand shillings (US$ 12.5) in the house was more than a year ago. I may be willing but I cannot afford.” (FGD 11–176, 36; old female non-members).

FGD participants expressed concerns about how the NHIF functioned, arguing that even when one wanted to become a member, it was difficult since the offices were located at district headquarters, leading to high transport costs.

“The problem that we have is that NHIF offices are far.…like the NHIF office here is in Nyeri [far town]. So you can see one has to pay transport to and from. If one was to make contributions every three months, transport costs end up being higher than the cost of the premium. If the offices were near here say Karatina [nearer town] a person can even walk to the offices to make payment or for other services.” (FGD 1–16, 11; old male members).

### Communities’ perceptions on the proposed national health insurance scheme and their preferred designs

**Figure 1:**
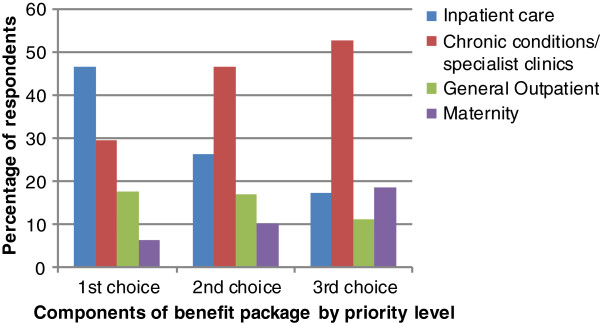
Preferred design of NHIS benefit package.

The majority of household survey respondents (93.0%) supported the implementation of a compulsory national health insurance scheme for Kenyans (Table 3). The government was the most preferred revenue -collecting and purchasing organization (51.8%) for such a scheme, while 32% preferred an autonomous purchasing agency, with some control from the government. Private purchasing institutions were hardly preferred (11.7%). Figure 1 shows preferences of different components of the benefit package. The results indicate that inpatient care was ranked as the number one priority; with 46.7% of the household survey respondents reporting that these services should be fully purchased by the NHIS should it come into existence. In contrast only 17.5% of respondents ranked outpatient services as their number one priority. Chronic conditions and specialised clinics were given first priority by 29.6% of respondents, while maternity care was only ranked first by 6.2% of respondents.

**Table 3:** Willingness to join a NHIS and preferred design features

**Variable**	**Number (%)**
Support for implementation of compulsory NHIS	
• Strongly support	304 (51.4)
• Support	246 (41.6)
• Oppose	27 (4.6)
• Strongly oppose	14 (2.4)
Total	591 (100)
Preferred revenue collecting organization	
• Public	306 (51.8)
• Private	69 (11.7)
• A combination of public and private features	189 (32.0)
• Don’t know	27 (4.6)
Total	591 (100)
Design of NHIS contribution rates	
• All Kenyans should pay equal amounts	102 (17.3)
• The rich should pay more than the poor	275 (46.5)
• The poor should not pay at all	205 (34.7)
• Don’t know	9 (1.5)
Total	591 (100)
Willing to join NHIS	
• Yes	506 (85.6)
• No	69 (11.7)
• Don’t know	16 (2.7)
Total	591 (100)
Willing to make contributions to support health care for the poorest Kenyans	
• Yes	522 (88.3)
• No	56 (9.5)
• Don’t know	13 (2.2)
Total	591 (100)
Reasons for wanting to join NHIS	
• Cheap way to access care	127 (21.5)
• Free health care for all Kenyans	343 (58.0)
• Comprehensive benefit package	43 (7.3)
• Compulsory	41 (6.9)
• Other	37 (6.3)
Total	591 (100)

FGD participants reported that they would join the NHIS for various reasons including: to be supported during the period of illness (i.e. cross-subsidisation), to ensure that they are protected since the costs of illness are unpredictable; to access high quality health care promptly and the fact that a NHIS would contract more health care providers than CBHIs, thus better choice of health service providers. Others reported that unlike private providers, who were motivated by profit, and who sometimes compromise patients' welfare by providing incorrect treatment, government facilities contracted under the NHIS were unlikely to over-diagnose patients.

On overall design, FGDs revealed that the community would prefer a NHIS that provided comprehensive inpatient and outpatient services, and should include all costs associated with treatment including drugs, surgery and ambulance. Before implementing the NHIS, it was reported that the government should work towards improving quality of services in public health facilities to ensure a constant supply of medicines and equipment, adequate personnel and reduced waiting time. Also emphasised was the need to have good interpersonal skills among health workers to ensure that people are treated with respect and dignity and to eliminate corruption.

“The basics….they should treat people with dignity and provide all required treatment. The contribution rate would not matter much as long as they treat people well and tackle corruption because that is what has led to bad services.” (FGD 2–36, 36; young male members).

“Another thing is that you may find a person who is admitted being told to go and buy drugs outside the facility while another person is being provided drugs in the same hospital. That corruption should be addressed.” (FGD 9–159, 28; young female members).

Close to half of the household survey respondents (46.5%) favored a progressive contribution structure, where the richest contributed a larger share of their income towards an NHIS (Table 3). Only 34.7% were of the opinion that the poorest population should be exempted from contributing towards a NHIS, while 17.3% preferred everyone to contribute equal amounts. Results from FGDs showed that the community would want to have their preferences considered in the NHIS design, particularly issues regarding contribution rates and benefits package in order to ensure that the set rates were affordable to the majority of the population and that the needed services were provided. However, caution was expressed regarding setting contribution rates too low at a rate that would make service provision difficult. Other suggestions included setting rates according to income levels in society, ensuring that the rich cross-subsidise the poor; the government should use taxes to fund the scheme and should it be on a contributory basis, the poorest population should be subsidised through tax funding.

“When the former health minister proposed that we pay ten shillings (US$ 0.1) in dispensaries, some of the facilities including this one here had to be closed because the amount requested was too low to support the services. The laboratory technologist is employed by the facility committee and the KES 10 (US$ 0.1) was unable to sustain his salary. So I would suggest we give a figure that can be able to sustain the provision of services at our facilities.” (FGD 8–143, 39; old male members).

Alternatively, the government can fund health through CBHIs. This would mean forming CBHIs where none exist nationally. These CBHIs would be represented in a committee in order to decide on the rates.” (FGD 1–14, 10; old male members).

“The government has a lot of money from tax. There is indirect tax… If we are taxed and it is used [for health care] just as it is used to build roads, by the water ministry and for other services, the Ministry of Health should provide free medical treatment using indirect taxes.” (FGD 1–15, 33; old male members).

“You know the government should also have its share [meaning subsidise the NHIS] because it taxes us. Here [by contributing] we are just helping the government so that we are able to help each other.” (FGD 12–201, 5; young male members).

## Discussion

This study set to explore communities’ understanding of health insurance, their perceptions of a future NHIS in Kenya and preferred design features. This section discusses the main findings and their implications on the efforts to transform the Kenyan health system financing towards universal health coverage.

Membership of health insurance schemes in the communities under study was quite high. More than half of the households had at least one member in health insurance scheme, while 41% of all individuals had insurance cover. These figures are significantly higher than national health insurance coverage levels, which are estimated as 10% of the population. High insurance coverage in the study area can be attributed to the fact that the study was conducted in a rural area with a long history of CBHI schemes, and which records the highest level of CBHI membership in Kenya. Consequently these results should be interpreted with caution since the study community had engaged with health insurance scheme for a long time and were more likely to be members of health insurance schemes compared to the rest of the Kenyan population.

The findings have demonstrated that there was some level of understanding of health insurance due to experiences working with CBHIs, but key concepts and how health insurance functions were not well understood. The majority of participants were aware that health insurance addresses the financial difficulties related to seeking health care, but it was not always clear why health insurance schemes operated differently from merry-go-rounds (rotating savings group). For example, there was very limited understanding of risk - pooling and cross-subsidization. The community hardly understood why contributions could not be refunded by the end of the financial year or forwarded to the next year’s contributions if they or their dependants had not fallen ill. Failure to do this was viewed by many as a loss, more so because these contributions did not attract any interests. Income cross-subsidisation was relatively well understood, with the majority of the population expressing their willingness and importance of the well off in society to subsidise contributions by the poorest groups. This could be associated with the fact that solidarity was common in the community, with people often coming together to help relatives and neighbours in cases of large hospital bills or in the event of death. However, there were concerns that even where the rich subsidised the poor, they were more likely to benefit from health services than the poor. The fact that health insurance is not well understood is not new. A recent study in South Africa reported similar findings, where only 53% of respondents understood the concept of risk - pooling, compared to 62% who were in favour of a system where the rich subsidised health care contributions for the poor [20]. An important factor that is likely to influence future implementation of a NHIS in Kenya is the communities’ acceptance of change. People are likely to accept something if they understood key concepts and how they work. As the country continues to find solutions to the ailing health system and the appropriate design of a NHIS, it is important that the community, who are the major beneficiaries of change, are sensitised and engaged to promote awareness, understanding of key concepts and their application. The presence of CBHIs in the study setting played a big role in people’s understanding of health insurance. Considering that CBHIs are not widespread in Kenya and only cover 1.2% of the population, it is important that efforts to engage the public go hand in hand with the design of health financing policies to ensure that the same are acceptable to the population, come implementation.

According to the community, membership of health insurance schemes was for the rich, the old and the sickly. None of the CBHIs operating in the study setting had mechanisms to waive premiums for the poor or destitute in society. This means that CBHIs discriminate against the poor, have significant implications for risk- pooling and sustainability of insurance schemes. A recent review of the literature identifies similar weaknesses associated with CBHIs in low income countries [21,22]. Nonetheless, CBHIs continue to be regarded as important avenues for UHC in many African settings. In Kenya, for example, the draft national health financing strategy emphasises the need to promote CBHIs in Kenya as part of financing mechanism for UHC [23]. In the financing strategy, membership of health insurance schemes will be compulsory for all Kenyans. CBHIs are being considered as the financing mechanisms for the informal sector workers, while formal sector workers will have their own pool under the National Health Insurance Scheme. Should this approach be adopted, the government must be willing to subsidise and support CBHIs to ensure that they are well designed, attract large numbers to allow for risk-pooling and subsidise membership for the poor. Most important is to allow for risk equalisation among pools to help address problems of bankruptcy for CBHIs that attract high risk individuals.

It was very clear that there is wide dissatisfaction with the current public health system. Concerns were expressed about quality of care, particularly related to availability of drugs, patient- provider interactions, long waiting times and discrimination against CBHIs members. However, it was not clear the extent to which these negative opinions were based on recent personal experiences or historical issues in the public health system. These concerns, it was reported, would have to be addressed for people to gain confidence in the public health system and in so doing contribute towards a NHIS. Similar findings were reported in Ghana, where the insured population reported waiting longer at health facilities than the non-insured and being discriminated by providers, receiving low quality drugs or being asked to buy them at private pharmacies, thereby incurring additional costs, and being subjected to verbal abuse [24,25]. Negative perceptions impact on trust in the public health system and hinder progress towards universal health coverage. In Ghana, dropouts of the national health insurance scheme gave poor experiences with the public health system as a major factor that contributed to their decisions of not renewing their membership [24]. It is important that the concerns raised regarding poor quality of care in Kenya, particularly in the public sector are addressed before implementation of the NHIS. CBHIs working in these areas should also note the concerns related to discrimination and work closely with health workers to ensure that their members are not discriminated. Experiences reported elsewhere suggest that discrimination could be due to many factors including cumbersome claiming process on the side of health facility, often leading to long gaps between providing services and payment; long administrative procedures, meaning that scheme members take longer to be attended to as their names have to be searched in databases that are often not in a user friendly manner [24].

Regarding benefits package, it was clear that people preferred a comprehensive package that included both inpatient and outpatient services, although inpatient services were perceived to be more deserving compared to outpatient care. Despite the negative perceptions of quality of care in the public health system and the belief that private facilities offered better services, when it came to collecting revenue for a NHIS, the community clearly favoured a system where the government collected the revenue and purchased services on behalf of the population. Only a minority preferred a private institution to take up this role, even when the government took some control of such an organisation. This shows that people still trust the government to look after their interests compared to private institutions. These findings have important implications for the design of a NHIS in Kenya. The government should take advantage of this trust and improve the care in the public health system before embarking on the implementation of NHIS. Improving the public health system will be of major contribution towards acceptability of financing mechanisms (health insurance and tax funding) for universal health coverage. Nonetheless, additional wok is needed to inform the design of UHC reforms in Kenya, including assessing the range of service entitlements that would be accessible and affordable to all Kenyans and which are implemented in a sustainable way.

Affordability of premiums, timing of contributions and the extent to which the needs of the poorest population would be met under a contributory scheme were major issues of concerns for a NHIS design. Nonetheless, there was a general agreement that premiums should not be set too low, to an extent that they undermine provision of quality services. Access to cash in many rural areas and in the informal sector is seasonal and making sure that timing for making contributions correspond with peak seasons when people have access to most of their annual income could improve on affordability and sustainability of premiums. Affordability of health insurance premiums was central in Ghana, where community members reported that the premiums were too high and unaffordable to many [25]. Although the Kenyan policy discussions are more geared towards a contributory national health insurance scheme, participants expressed their preference for a tax - funded scheme, since all Kenyans pay taxes either directly or indirectly. A tax - funded health system was regarded as more inclusive compared to a NHIS, which was perceived to target the rich more than the poor. The design of health systems reforms in Kenya should also consider the possibility of achieving UHC through a predominantly tax - funded system.

### Limitations

This study was conducted in two settings with a strong presence of CBHIs. The community was therefore more exposed to health insurance concepts and to the NHIF compared to other settings in Kenya. It is possible that this exposure contributed significantly to their perceptions on health insurance and that these are likely to be different in other settings. However, the study still reported very limited understanding of health insurance, implying that much more education and sensitisation regarding health insurance is needed in settings without CBHIs. Thus the findings presented in this paper have important policy implications regardless of this limitation.

## Conclusions

This study has demonstrated that there was very limited understanding of health insurance in the communities under study, particularly related to the concept of risk- pooling. As the country continues to put mechanisms in place for UHC, it is important that communities are educated and engaged for them to understand the importance of risk-pooling and cross-subsidisation. Affordability of premiums was expressed as a major challenge to health insurance membership. The Kenya government needs to carefully consider the value of achieving universal health coverage through a contributory basis versus providing coverage for those working in the informal sector through tax funding. This will include assessing the mechanisms to increase tax revenue collection, through for example improving efficiency in revenue collection and introducing innovative taxes to support the health system. Finally, the perceived poor quality of care in public health systems can be a major hindrance of UHC. Good quality services, particularly related to drug availability and interpersonal relationships between clients and health providers can boost trust in the public system and in so doing encourage people to belong to health insurance. The feasibility of financing reforms for UHC, particularly those related to NHIS, will largely depend on the quality of care in the Kenyan public health system.

## Competing interests

The authors declare that they have no competing interests.

## Authors’ contributions

JC was responsible for the overall design of the study. JC and SM were involved in data collection, analysis and writing. DK supported data analysis and writing. All authors read and approved the manuscript.

## Pre-publication history

The pre-publication history for this paper can be accessed here:

http://www.biomedcentral.com/1472-6963/13/474/prepub
